# Variations in Strain Distribution at Distal Radius under Different Loading Conditions

**DOI:** 10.3390/life12050740

**Published:** 2022-05-16

**Authors:** Jonas A. Pramudita, Wataru Hiroki, Takuya Yoda, Yuji Tanabe

**Affiliations:** 1 College of Engineering, Nihon University, Koriyama 963-8642, Japan; 2 Graduate School of Science and Technology, Niigata University, Niigata 950-2181, Japan; 3 Graduate School of Medical and Dental Sciences, Niigata University, Niigata 950-2181, Japan; takuyayo@med.niigata-u.ac.jp; 4 Management Strategy Section, President Office, Niigata University, Niigata 950-2181, Japan; y.tanabe@eng.niigata-u.ac.jp

**Keywords:** distal radius fracture, fall, loading direction, load distribution, strain distribution, finite element analysis, fracture pattern

## Abstract

Distal radial fractures exhibit various fracture patterns. By assuming that the strain distribution at the distal radius affects the diversification of the fracture pattern, a parameter study using the finite element model of a wrist developed from computed tomography (CT) images was performed under different loading conditions. The finite element model of the wrist consisted of the radius, ulna, scaphoid, lunate, triquetrum, and major carpal ligaments. The material properties of the bone models were assigned on the basis of the Hounsfield Unit (HU) values of the CT images. An impact load was applied to the scaphoid, lunate, and triquetrum to simulate boundary conditions during fall accidents. This study considered nine different loading conditions that combine three different loading directions and three different load distribution ratios. According to the analysis results, the strain distribution at the distal radius changed with respect to the change in the loading condition. High strain concentration occurred in regions where distal radius fractures are commonly developed. The direction and distribution of the load acting on the radius were considered to be factors that may cause variations in the fracture pattern of distal radius fractures.

## 1. Introduction

Distal radius fracture due to fall occurs frequently in the elderly population [1], especially in people with osteoporosis [2,3]. In Japan, 108.6 incidence of distal radius fractures per 100,000 persons was confirmed in Sado City, Niigata, in 2004 [4]. In addition, another study reported that 514 incidence of distal radius fractures per 100,000 persons occurred in Sakaiminato, Tottori between 2010 and 2012 [5]. The number of distal radius fracture incidence increases annually because of the effect of population aging [6].

**Figure 1:**
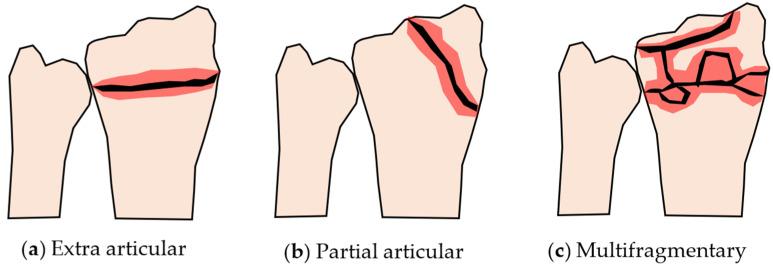
Some typical distal radius fracture patterns. (**a**) Simple extra articular fracture, (**b**) simple partial articular fracture, (**c**) multifragmentary complete articular fracture.

Distal radius fractures are considered ‘less severe’ when compared to spine fractures or femoral neck fractures because spine and femoral neck fractures demand bed rest of patients [7,8]. However, a unique characteristic of the distal radius fracture is the variation in the fracture pattern [9], according to which the fracture can be divided into Colles’ fracture, Smith’s fracture, and Barton’s fracture [10]. In Colles’ fracture, the broken distal end of the radius is displaced dorsally. On the other hand, in Smith’s fracture, the broken distal end of the radius is displaced volarly. Furthermore, Barton’s fracture is an articular fracture in which the broken distal end of the radius involves the radiocarpal joint. Additionally, a more detailed classification of distal radius fractures, called AO classification [11,12], shows nine types of fracture patterns. The AO classification combines extra articular, partial articular, and complete articular fractures with simple, complex, and multifragmentary fractures. The patterns of some typical distal radius fractures are illustrated in Figure 1. Based on this classification, the difficulty level of the treatment and the ease of recovery of each type of fracture can be easily predicted.

Although the exact reason for the variation in the fracture pattern of distal radius fractures remains unknown, several factors are presumed to be the cause of this variation. Internal factors, such as bone mineral density (BMD), bone quality, and bone or articular geometry, as well as the external factors, such as load characteristics, constraint condition, and forearm posture, can affect the fracture pattern. Some clinical studies have investigated the effect of these factors on fracture patterns. Investigation of the relationship between BMD and distal radius fracture shows that, although BMD may be related to the presence of fracture, it cannot be used as a predictor of the fracture pattern [13,14,15]. Moreover, an investigation on the effect of bone shape on scaphoid fractures also revealed that there is a relationship between the bone shape and the presence of fractures [16]. However, no connection between the bone shape and fracture patterns could be drawn from the study. According to these studies, we assumed that the internal factors have less effect on the fracture pattern when compared to external factors. Unfortunately, engineering studies on distal radius fractures are few in number, and the contribution of external factors to fracture patterns has not been thoroughly investigated.

Engineering studies on distal radius fractures can be divided into experimental and numerical analyses. Experimental studies generally use a part of a cadaver’s forearm or a single cadaver’s radius as the specimen. Burkhart et al. [17], Matsuura et al. [18], Johnson et al. [19], and Zapata et al. [20] conducted quasi-static compression tests using a cadaver’s forearm or radius and determined the fracture load or strain distribution on the distal radius. The cadaver test is relatively expensive, and it is difficult to conduct many tests considering different boundary conditions because of the limited number of specimens that can be acquired. Moreover, it is not easy to determine the stress and strain distribution at the distal radius, which is believed to be related to the variations in the fracture pattern. Numerical analysis was typically performed using the finite element method (FEM). Edwards et al. [21], Matsuura et al. [22], Mancuso et al. [23], and Revel et al. [24] conducted finite element analysis (FEA) using finite element models of the radius or wrist bones. The simulation quantitatively evaluated the stress and strain at the distal radius, and the results were considered reasonable when compared to the results obtained from the experimental study. Based on the above studies, we considered FEA as the best tool to investigate the contribution of external factors, including the mechanical conditions during fall, in generating different fracture patterns, because we can easily change the boundary conditions of the simulation. Furthermore, different boundary conditions can be investigated both easily and at a low cost compared to conventional experimental studies [25].

In this study, assuming that the strain distribution at the distal radius affects the type of the fracture, we developed a finite element model of the wrist from a typical computed tomography (CT) data of an elderly female and investigated the effect of dynamic loading conditions on the strain distribution at the distal radius using the finite element model. The results of the analysis were expected to provide a basis for understanding the variations in fracture patterns in distal radius fractures.

## 2. Materials and Methods

### 2.1. CT Data

**Figure 2:**
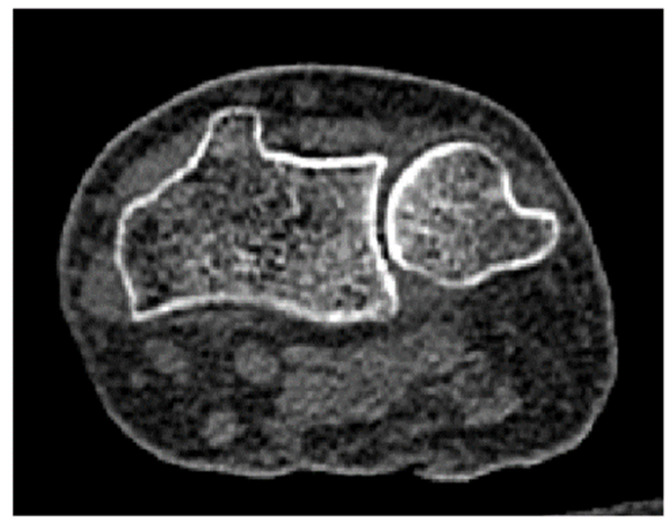
A CT image (512 × 512 pixels) of subject’s wrist showing the radius and ulna. Seventy slices of CT images were utilized in the present study.

A finite element model of the wrist was constructed from the CT data of the right forearm of a patient at Uonuma Kikan Hospital, Niigata, Japan. The patient was a 76-year-old female with a body weight of 51.6 kg. The scan was performed in a fully extended wrist position. The CT images (Figure 2) were of 512 × 512 pixels, with a slice thickness of 1 mm. The research protocol, including the use of CT data, was approved by the ethics committee of Niigata University, Niigata, Japan.

### 2.2. Three-Dimensional Modeling

**Figure 3:**
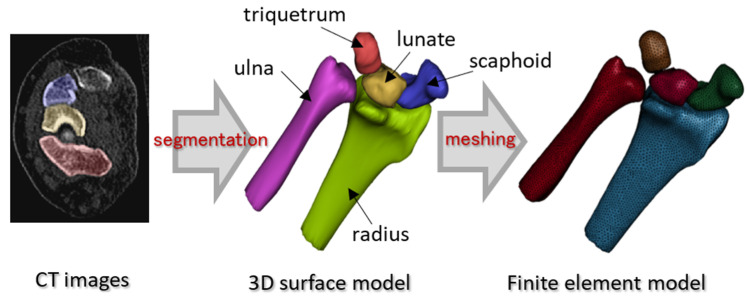
Construction procedure of bone finite element models. Finite element model was constructed from 3D surface model reconstructed based on CT images.

To construct a finite element model of the wrist, bone segmentation was performed using Mimics v20 (Materialise NV, Leuven, Belgium) on 70 slices of CT images. Three-dimensional models of the radius, ulna, triquetrum, scaphoid, and lunate were then reconstructed based on the segmentation results. The smoothing and fixing of the models were also performed prior to meshing using 3-Matic v14 (Materialise NV, Leuven, Belgium). Finally, meshing was performed using Hypermesh 2019 (Altair Engineering Inc., Troy, MI, USA). The procedure for the finite element modeling of bones is depicted in Figure 3.

The finite element models consisted of tetrahedron elements with an element size of 1 mm. The element size was chosen by considering the convergence of the result, the calculation time consumed for analysis using our workstation, and the fact that this element size provides a reasonable result according to several previous studies [21,26,27]. The number of nodes and elements were 42,661 and 218,392, respectively.

### 2.3. Bone Material Properties Assignment

**Figure 4:**
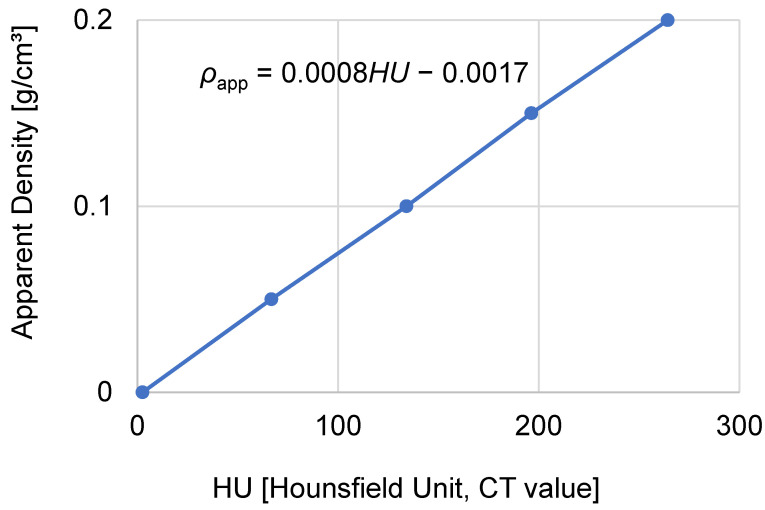
Relationship between the apparent density and HU of the bone phantom containing five hydroxyapatite cylinders. A linear regression was conducted to obtain the equation.

Bone material property distribution is believed to provide a unique mechanical response distribution in bones, which may vary the fracture pattern of distal radius fractures. Therefore, the density and Young’s modulus at a location were estimated from the Hounsfield unit (HU) of the bone area shown on the CT images. While performing the CT scan of the wrist, we simultaneously scanned a bone phantom (B-MAS200, Kyoto Kagaku Co. Ltd., Kyoto, Japan) consisting of five hydroxyapatite cylinders with different apparent densities [28]. The image of the bone phantom was thereafter analyzed using Mimics v20 (Materialise NV, Leuven, Belgium) to determine the HUs of the five hydroxyapatite cylinders inside the phantoms. Linear regression was conducted to determine the relationship between the apparent densities and HUs of the hydroxyapatite cylinders, as depicted in Figure 4. Using this relationship, the apparent density of the bone can be estimated based on the HU of the bone image.

Based on the estimated apparent density, the ash density was calculated using Equation (1). Furthermore, Young’s modulus was calculated based on the ash density using Equation (2). Here, $\rho_{app}$ is the apparent density, $\rho_{ash}$ is the ash density, and E is the Young’s modulus. These equations were adopted from literature [21,29]. The bone material properties (ash density and Young’s modulus) were then assigned to the model.

(1) $$\rho_{ash}=0.0698+0.839\rho_{app}$$

(2) $$E=10.5\rho_{ash}{}^{2.29}$$

**Figure 5:**
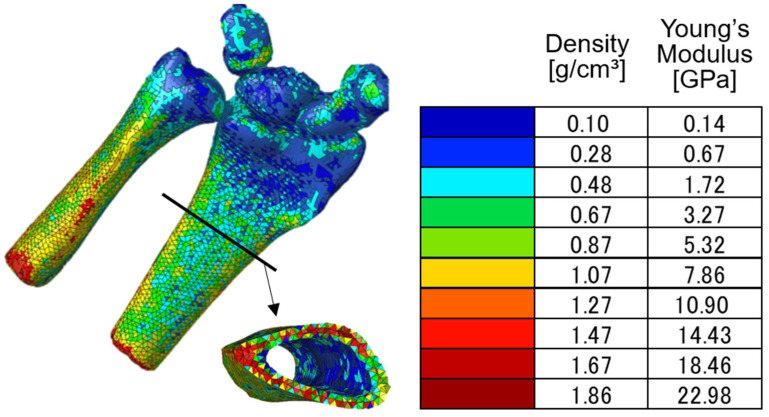
Bone model with assigned material properties calculated using Equations (1) and (2). The material properties were divided into 10 groups as represented by different colors.

We did not assign different material properties to each element in the model to avoid unnecessary complexity. In this study, we divided the material properties into 10 groups, as depicted in Figure 5. Material property assignment was performed using Mimics v20 (Materialize NV, Leuven, Belgium). Meanwhile, the Poisson’s ratio was assumed to be constant at 0.3 [30,31].

In addition, to determine the effect of nonuniform material properties on the mechanical response of the distal radius, we constructed the finite element model of a wrist with a uniform density of 1.5 g/cm^3^ and a uniform Young’s modulus of 15 GPa [32]. This model was used in the analysis under the same conditions as those of the nonuniform model.

### 2.4. Modeling of Ligaments and Cartilages

**Figure 6:**
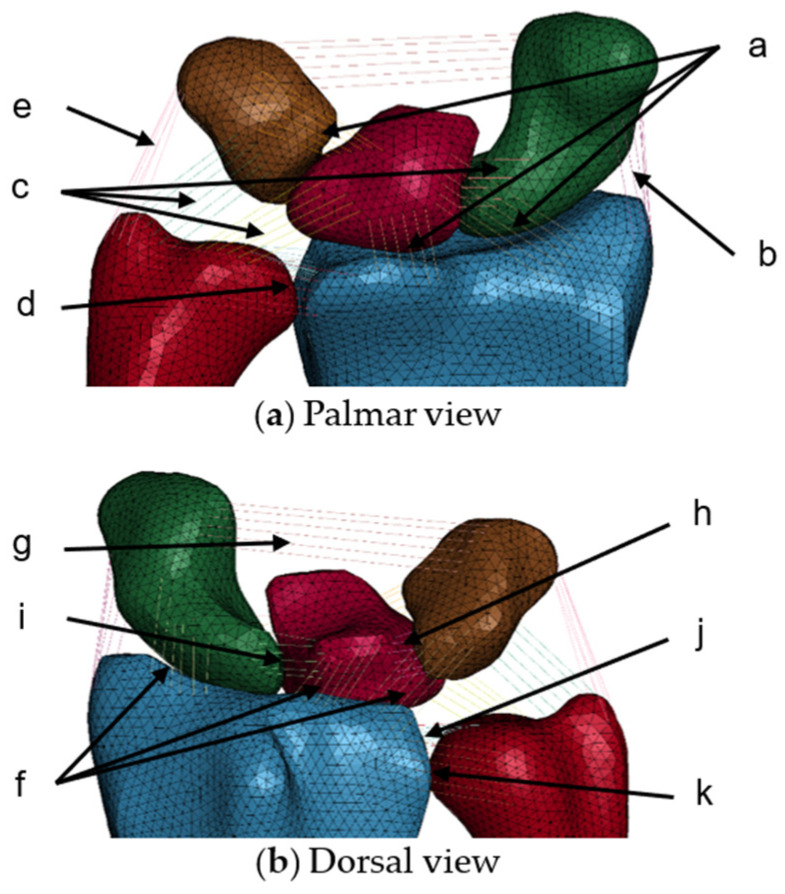
Eleven ligament models modeled with bar elements that constrained the five bones (radius, ulna, scaphoid, lunate, and triquetrum) in wrist finite element model. (**a**) Palmar view. (**b**) Dorsal view. The letters a to k shows the names of the ligaments listed in Table 1.

Ligaments were required to prevent lunate, triquetrum, and scaphoid dislocation during loading. Therefore, 11 ligaments that constrained five bones were selected from the literature [33] and modeled as bar elements. The ligament models consisted of six bars with elastic properties, as depicted in Figure 6. The origin and insertion of the ligaments were decided according to anatomy literature. Nodes at both ends of the ligament models were tied to the bones model. The material properties of these ligaments [34] are listed in Table 1.

In this study, cartilages were not modeled three-dimensionally to reduce computational cost. A previous study reported that the cartilage layer did not have a significant effect on the bone stress under impact loading [35]. However, to model the articulations and contact behavior among bones, the contact definition (automatic surface-to-surface contact) was set among the whole surface of the five bones with friction coefficients of 0.3 [36]. The contact areas were not constant, because it changed with the change in the loading condition.

### 2.5. Analysis Conditions

**Figure 7:**
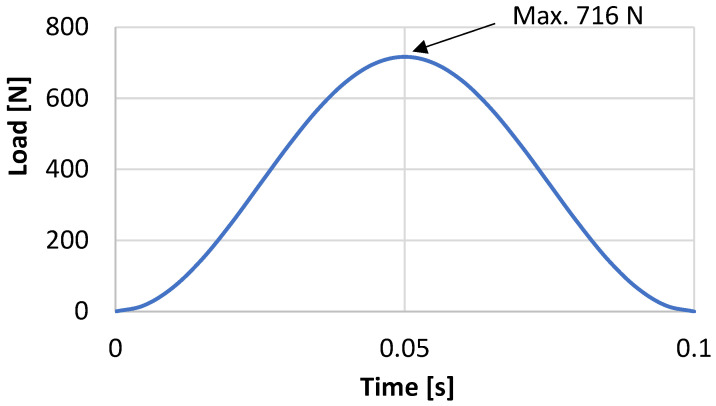
Load wave used in the fall accident simulation. The load wave was created based on the literature [37]. The maximum load was scaled according to the body weight of the subject.

An analysis was conducted to simulate the load on the palmar in a forward fall accident. The measurement results obtained from a human subject experiment [37] were used to generate a sinusoidal load wave, as depicted in Figure 7. A maximum load (W) of 716 N was determined by scaling the maximum load of 1000 N obtained from the experiment using the ratio of the volunteer’s body weight between the experiment and the wrist model (1:0.72).

**Figure 8:**
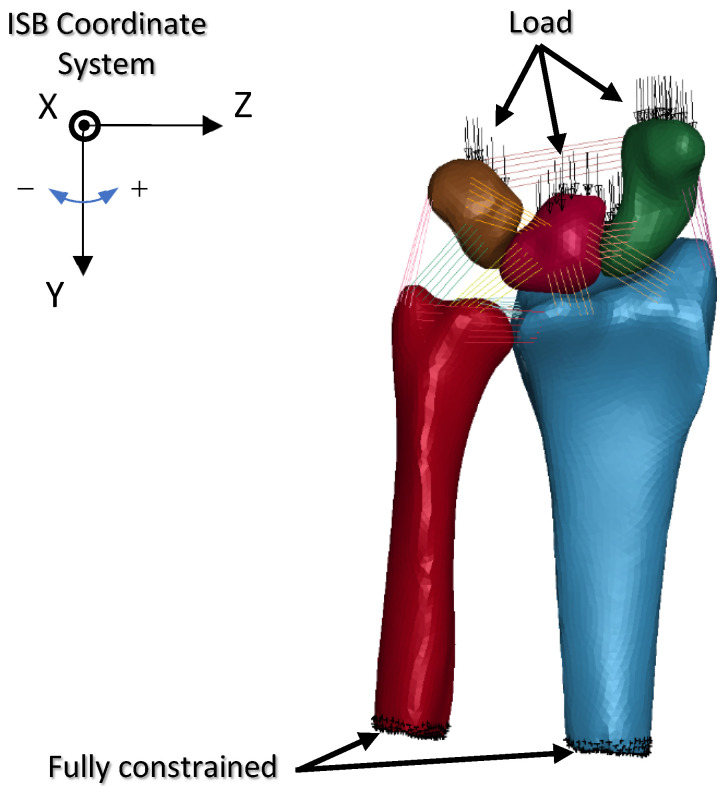
Constraint and load applied on the wrist finite element model. Load was distributed to scaphoid, lunate, and triquetrum as described in Table 2. Loading direction was configured as described in Table 3.

The finite element model of the wrist was fully constrained at the proximal end, which is a condition similar to that described in previous studies [18,21]. A distributed load was then applied to the scaphoid, lunate, and triquetrum along the Y-axis of the ISB radius co-ordinate system [38], as depicted in Figure 8. A parameter study was conducted by changing the load distribution ratio between the scaphoid and lunate-triquetrum, as well as the loading direction. The load distribution ratios and loading directions analyzed in this study are listed in Table 2 and Table 3, respectively. These conditions were assumed to represent the forearm posture during fall accidents, which may lean toward the radial or ulnar direction. Combinations of load distribution ratios and loading directions resulted in nine different analysis conditions.

The analysis was conducted using LS-DYNA R10 (Ansys Inc., Canonsburg, PA, USA) using a dynamic explicit method. The analysis results were then visualized using LS-PrePost 4.7 (Ansys Inc., Canonsburg, PA, USA) to determine the von Mises strain distribution at the distal radius.

## 3. Results

**Figure 9:**
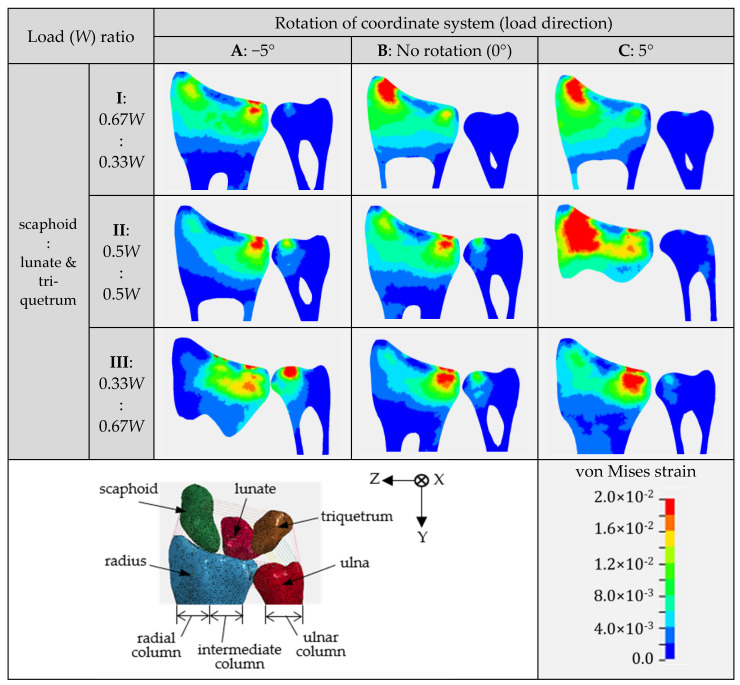
Von Mises strain distribution on a coronal cross-section plane of distal radius and distal ulna obtained from finite element analyses under nine different analysis conditions. A color bar shows the magnitude of the von Mises strains. Red area indicates a high strain concentration zone.

The results of the analysis are depicted in Figure 9. This figure compares the von Mises strain distribution on the frontal cross-sectional area of the distal radius and distal ulna at the maximum load obtained from finite element analyses. The row indicates the change in the strain distribution with respect to the change in the load distribution ratio. The column indicates the change in the strain distribution with respect to the change in the loading direction. The red areas indicate areas where strain concentration occurs.

## 4. Discussion

As depicted in Figure 9, the strain distribution varied with respect to the change in the loading condition. Strain concentration occurred in a region that was separated proximally from the cartilage surface. This tendency was also reported in previous studies [19,20,22]. The region with high strain showed low bone density and low Young’s modulus compared to the diaphyseal region, as indicated in Figure 5. Low bone density and low Young’s modulus caused this region to largely deform during loading, further generating high strain in the region. According to the high-strain-concentration area (red area) in the distal radius, the analysis results can be divided into two clusters. The first cluster is a high strain in the radial column, as shown in the analysis results of IB, IC, and IIC. The second cluster is the high strain in the intermediate column, as shown in the analysis results of IA, IIA, IIB, IIC, IIIA, IIIB, and IIIC. Additionally, a relatively higher strain concentration in the ulnar column could also be observed from the analysis results of IIA, IIB, IIIA, and IIIB.

Under condition II, where the load was equally distributed to the scaphoid and lunate-triquetrum, the strain increased significantly in the radial column with a change in the loading direction from −5° to 5°. A loading direction of 5° was parallel to the normal direction of the scaphoid facet surface, which caused high compression of the scaphoid to the facet surface. This compression is considered to cause a high strain concentration in the radial column. This phenomenon could also be confirmed under condition I, where the scaphoid was subjected to a higher load compared to the other bones, despite no significant change in strain between the loading directions of 0° and 5°. On the other hand, under condition III, where the lunate and triquetrum were subjected to a higher load compared to the scaphoid, the change in the loading direction did not cause a significant increase in the strain at the radial column. According to this result, we can see that the strain at the distal radius was affected not only by the loading direction, but also by the load distribution ratio applied to it.

A high strain at the intermediate column can be seen from the majority of the analysis results, regardless of the loading direction and load distribution ratio. This result indicates that the load is always transmitted at a significant magnitude to the intermediate column under all the loading conditions adopted in this study. A high strain at the ulnar column could be seen from the analysis results under a load direction of −5° combined with a larger load applied to the lunate and triquetrum. A loading direction of −5° is considered to compress the radius to the ulna, further generating a high strain on the distal ulna, owing to the contact with the sigmoid notch of the radius. Furthermore, under this loading direction, the scaphoid was considered to slide on the oblique facet surface, further mitigating the high strain concentration in the radial column. High strains in the radial and intermediate columns were caused by load transmission, owing to the contact with the scaphoid and lunate, respectively. Marquez-Florez et al. [33] reported that more than 85% of the applied load was transmitted to the radius via the scaphoid and ulna. According to this report, the radius is subjected to a higher load than the ulna, which is in good agreement with the results of the present study.

Assuming that high strain causes fracture, condition IIC may generate multifragmentary intra-articular fractures, whereas conditions IIA or IIB may generate partial articular fractures. Furthermore, condition IIB may generate extra articular fractures in the region, which is the boundary between the high- and low-strain areas. However, although high strain is strongly related to fracture occurrence, it should be noted that the high-strain area and fracture site might be different. In the future, damage material models should be introduced to simulate the development of fractures more precisely.

**Figure 10:**
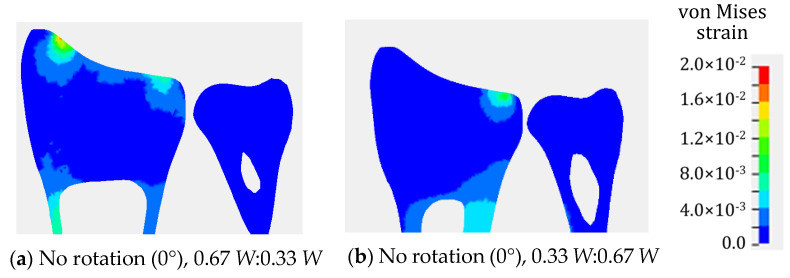
Von Mises strain distribution in distal radius and distal ulna obtained from finite element analyses using a finite element model with uniform material properties. Load on scaphoid: lunate and triquetrum was 0.67 *W*:0.33 *W* for (**a**), and 0.33 *W*:0.67 *W* for (**b**). No rotation of ISB co-ordinate system. A contour legend shows the magnitude of the von Mises strains. Red area indicates a high-strain-concentration zone.

As explained above, the strain concentration is related to the distribution of bone density and Young’s modulus in the distal radius. To check the effect of nonuniform material properties on the strain distribution, a comparison with the analysis results using uniform material properties was conducted. Figure 10 depicts the von Mises strain distribution obtained from the wrist model with uniform material properties under conditions IB and IIIB. According to the results depicted in Figure 9 and Figure 10, the strain concentration was completely different. The wrist model with uniform material properties could not simulate the high-strain-concentration region at the distal radius. Therefore, it is considered inappropriate to use a finite element model with uniform material properties for the analysis of distal radius fractures.

The present study has several limitations. Because the purpose of this study was to determine the strain distribution at the distal radius, we only conducted a comparison of the strain distribution pattern. We assumed that the result should not be evaluated quantitatively because the model was not validated and verified against cadaver tests, as performed in previous similar studies [19,39,40,41]. However, as described above, the tendency of the strain distribution was consistent with previously reported results. In addition, the maximum von Mises strain obtained in this study was approximately 2%. This value was almost the same as the result of a study conducted by Zapata et al. [20] under dynamic loading conditions, further indicating that the strain value is considered reasonable.

The results of this study were believed to provide basic knowledge of the mechanism of variations in distal radius fracture. Moreover, it can be used to predict the condition of a fall accident that generated a specific distal radius fracture. To the best of author knowledge, our study is the first numerical study on the effect of load distribution and loading direction on the mechanical response of distal radius. In the future, we plan to validate the finite element analysis result against experimental data.

## 5. Conclusions

We successfully conducted a numerical study using the finite element model of a wrist simulating a fall on a stretched hand under different loading directions and load distribution ratios. The strain distribution at the distal radius varied with respect to the variations in the loading direction and load distribution ratio, owing to the alteration of the contact situation between the scaphoid and radius, as well as between the lunate and radius. In addition, we confirmed a high strain concentration in the region where a distal radius fracture is likely to occur. The direction and distribution of the impact load acting on the radius are believed to have some effect on the diversification of the fracture pattern in distal radius fractures.

## Figures and Tables

**Table 1:** Material properties of the ligament models adopted from the literature [34].

Ligament	Density (g/cm^3^)	Young’s Modulus (MPa)	Poisson’s Ratio
a: Palmar radiocarpal	1.2	26.9	0.3
b: Radial collateral		22.6	
c: Palmar ulnocarpal		22.6	
d: Palmar radioulnar		22.6	
e: Ulnar collateral		22.6	
f: Dorsal radiocarpal		119.3	
g: Dorsal intercarpal		47.5	
h: Dorsal lunotriquetral		22.6	
i: Dorsal scapholunate		22.6	
j: Triangular fibrocartilage complex		22.6	
k: Dorsal radioulnar		22.6	

**Table 2:** Condition I to III shows load (*W*) distribution ratios.

Condition	Lunate and Triquetrum	Scaphoid
I	0.67 *W*	0.33 *W*
II	0.5 *W*	0.5 *W*
III	0.33 *W*	0.67 *W*

**Table 3:** Condition A to C shows loading directions along Y-axis of ISB co-ordinate system.

Condition	ISB Co-Ordinate System
A	−5° rotation around X-axis
B	No rotation (0°)
C	5° rotation around X-axis

## Data Availability

Not applicable.
